# Cortical signature of patients with HBV-related cirrhosis without overt hepatic encephalopathy: a morphometric analysis

**DOI:** 10.3389/fnana.2015.00082

**Published:** 2015-06-08

**Authors:** Xiu Wu, Xiao-Fei Lv, Yu-Ling Zhang, Hua-Wang Wu, Pei-Qiang Cai, Ying-Wei Qiu, Xue-Lin Zhang, Gui-Hua Jiang

**Affiliations:** ^1^Key Laboratory for NeuroInformation of Ministry of Education, School of Life Science and Technology, University of Electronic Science and Technology of ChinaChengdu, China; ^2^Department of Medical Imaging, Sun Yat-sen University Cancer Center, State Key Laboratory of Oncology in South China, Collaborative Innovation Center for Cancer MedicineGuangzhou, China; ^3^Department of Medical imaging, Guangzhou Brain Hospital, The Affiliated Hospital of Guangzhou Medical UniversityGuangzhou, China; ^4^Department of Medical Imaging, Guangdong No. 2 Provincial People’s HospitalGuangzhou, China; ^5^Medical Imaging Centre, Nanfang Hospital, Southern Medial UniversityGuangzhou, China

**Keywords:** cirrhotic patients, HBV, cortical thickness, cortical gyrification, brain edema

## Abstract

Previous studies have shown that patients with hepatitis B virus-related cirrhosis (HBV-RC) without overt hepatic encephalopathy (OHE) are associated with a varying degree of cognitive dysfunction. Several resting-state functional magnetic resonance imaging (fMRI) studies have been conducted to explore the neural correlates of such cognitive deficits, whereas little effort has been made to investigate the cortical integrity in cirrhotic patients without OHE. Here, using cortical thickness, surface area and local gyrification index (lGI), this study performed a comprehensive analysis on the cortical morphometry of patients with HBV-RC without OHE (HBV-RC-NOHE) vs. matched healthy controls. Compared with healthy controls, we found significantly increased cortical thickness in the bilateral lingual and parahippocampal gyrus, right posterior cingulate cortex, precuneus, peri-calcarine sulcus and fusiform gyrus in patient with HBV-RC-NOHE, which may closely relate to be the low-grade brain edema. Cortical gyrification analysis showed significantly increased lGI in the left superior and inferior parietal cortex as well as lateral occipital cortex, which was speculated to be associated with disruptions in white matter connectivity and sub-optimal intra-cortical organization. In addition, the mean cortical thickness/lGI of the regions with structural abnormalities was shown to be negatively correlated with psychometric hepatic encephalopathy score (PHES) of the patients with HBV-RC-NOHE. These morphological changes may serve as potential markers for the preclinical diagnosis and progression of HBV-RC-NOHE.

## Introduction

Hepatitis B virus-related cirrhosis (HBV-RC) is one of the most serious public health problems in Asia, which is associated with high infection, morbidity and mortality rates (Moriwaki et al., 2010). Hepatic encephalopathy (HE) is the most common neuropsychiatric complication in HBV-RC and is characterized by a wide spectrum of clinical manifestations that range from mild cognitive impairment to coma and death (Iwasa et al., 2012). Clinically, overt hepatic encephalopathy (OHE) could be easily identified in cirrhotic patients by the appearance of neuropsychiatric symptoms (Córdoba, 2011). In contrast, cirrhotic patients without OHE do not present recognizable clinical neuropsychiatric symptoms but associate with a varying degree of cognitive deficits mainly in visual perception, visuo-constructive abilities, fine motor performance, attention and memory (Lv et al., 2013a), which could be quantitatively measured by the psychometric hepatic encephalopathy score (PHES; Weissenborn, 2008). Using resting-state functional magnetic resonance imaging (fMRI) data, several studies have been conducted to explore the neural correlates of the cognitive deficits in cirrhotic patients without OHE (Lv et al., 2013a,b; Chen et al., 2014). Nevertheless, little effort has been made to investigate the cortical integrity in cirrhotic patients without OHE.

Since the advent of magnetic resonance imaging (MRI), various morphometric approaches have been developed to identify macroscopic changes in the human brain. In cirrhotic patients, most of existing morphometric studies were performed by using voxel-based morphometry (VBM; Chen et al., 2012; Iwasa et al., 2012). However, the interpretation of VBM results can be difficult given that an actual physical characteristic is not measured directly (Singh et al., 2006). Moreover, the specific contribution of the anatomical properties of the cortical mantle to these results remains unknown since VBM provides a mixed measure of cortical gray matter (GM) including cortical thickness, cortical surface area and/or cortical folding (Palaniyappan and Liddle, 2012). Current surface-based morphometry allows us to quantitatively investigate such physical properties using cortical thickness, surface area, and local gyrification index (lGI; Schaer et al., 2008; Palaniyappan and Liddle, 2012). To our knowledge, only one study documented cortical thickness changes in superior temporal cortex and precuneus in patients with minimal hepatic encephalopathy (MHE; Montoliu et al., 2012). Nevertheless, the interpretation of the results should be cautious since the sample of their study included MHE patients of various causes and lacks homogeneity. Additionally, the patterns of surface area and cortical folding, another two morphometric characteristics that are thought to be both genetically and phenotypically independent of cortical thickness (Panizzon et al., 2009; Hogstrom et al., 2013), have never been explored in cirrhotic patients without OHE.

Using cortical thickness, surface area and lGI, this study aims to perform a comprehensive analysis on the cortical morphometry of a relatively homogeneous sample of patients with HBV-RC without OHE (HBV-RC-NOHE) vs. matched healthy controls. Based on previous studies (Montoliu et al., 2012; Lv et al., 2013a,b), we hypothesized that patients with HBV-RC-NOHE would exhibit cortical thickness reduction in multiple brain regions including the parietal lobe, the temporal lobe and the occipital lobe. In addition, given that many studies have reported intensive white matter abnormalities (Kumar et al., 2008; Chen et al., 2012; Qi et al., 2013) and the hypothesis that cortical gyrification was mainly caused by the tension of the underlying white matter connectivity (Van Essen, 1997; Herculano-Houzel et al., 2010), cortical gyrification alterations are also expected in patients with HBV-RC-NOHE.

## Materials and Methods

### Subjects

Twenty-six patients (23 male; mean age, 45.85 ± 9.0 years; age range, 32~67 years) with chronic liver cirrhosis caused by HBV infection were included in this study. All patients had HBV-RC diagnosed by biopsy or on the basis of case history, clinical examination, biochemical and imaging findings. OHE was diagnosed when the West Haven criteria indicated stage I disease or higher. Patients were excluded if they had current symptoms of OHE at the time of recruitment, any history of OHE, other types of viral hepatitis, gastrointestinal hemorrhage or bacterial infection (within 1 month before the study), a transjugular intrahepatic portosystemic or a surgical portocaval shunt, diffuse hepatocellular carcinoma, or were taking drugs that could alter cerebral function. All patients underwent a detailed clinical examination. The severity of liver disease was determined according to the Child-Pugh score.

For comparison, 22 healthy controls (19 male; mean age, 46.3 ± 9.8 years; age range, 28~63 years) matched for age, sex and years of education, without disease of liver and other systems, were recruited via advertising within the hospital. All controls received detailed clinical and neurological examinations on the same day as the MRI scans. Exclusion criteria for all patients and controls included age lower than 18 or greater than 70 years, alcoholism, neurological or psychiatric diseases, a history of substantial head trauma that resulted in unconsciousness or at least in substantially altered mental status, infection with human immunodeficiency virus, hypertension, diabetes, poor vision, other major medical illness, left-handedness, and any focal abnormality detected on routine brain MRI examinations.

This study was approved by the Medical Research Ethics Committee of Nanfang Hospital, Southern Medical University. Written informed consent was obtained from all the participants before the study. Detailed clinical and demographic data of patients with HBV-RC-NOHE and healthy controls are shown in Table 1.

**Table 1:** **Clinical and demographic data of the participants**.

Characteristic	Cirrhotic patients (*n* = 26)	Healthy controls (*n* = 22)
Age (years)	45.85 ± 9.0	46.3 ± 9.8
Sex ratio, M/F	23/3	19/3
Education (years)	9.81 ± 4.45	11.73 ± 2.86
Child–Pugh A/B/C	13/8/5	–
Total serum bilirubin (IU/L)	93.56 ± 178.03	N/A
Serum albumin (g/L)	34.91 ± 7.03	N/A
Prothrombin time (s)	16.32 ± 2.87	N/A
PHES*	−3 (−9~1)	0 (−2~3)

Note: *indicates significant between-group difference (p < 0.001). N/A = not available.

### Neuropsychological Tests

The PHES battery, a psychometric test that involves visual perception, construction, visual/spatial orientation, motor speed and accuracy, concentration, and attention (Weissenborn, 2008), was completed to all the subjects to quantitatively measure their cognitive performance. This battery contains five paper-and-pencil psychometric tests, including the number connection test A (NCT-A, in seconds), and the modified number connection test B (NCT-B, in seconds), the digit symbol test (DST, in numbers), the serial dotting test (SDT, in seconds) and the line tracing test (LTT, the sum of time taken in seconds and the number of errors). All subjects completed the test battery after an appropriate explanation and demonstration. The PHES value could range from +5 to −15, with lower value corresponding to worse cognitive performance. The exact method for the calculation of PHES values could be found in details in a previous study (Lv et al., 2013a).

### MRI Data Acquisition

MRI data were obtained on a Philips Achieva 1.5-T Nova Dual MR scanner. Each subject lay supine with the head snugly fixed by a belt and foam pads. A three-dimensional fast field echo (FFE) pulse sequence was used to produce 155 1-mm-thick contiguous axial images [time to echo (TE) = 4.1 ms, repetition time (TR) = 25 ms, matrix = 256 × 256, field of view (FOV) = 23 cm, flip angle = 30°, number of excitation: 1].

### Image Preprocessing

Each scan was processed using *Freesurfer*^1^ with its volume and surface pipeline (Dale et al., 1999; Fischl et al., 1999a). Starting from the segmentation of white matter and the tessellation of the gray/white matter boundary, an initial surface was obtained after automated topological correction. This surface was used as the initial shape for the deformable model that was used to reconstruct the pial surface. Both surfaces were represented by points and triangles composed of the points. Please note that the points at the gray/white matter surface had a one-to-one correspondence with the points at the pial surface.

### Cortical Thickness and Surface Area

Cortical thickness was defined at each point on the pial surface (as well as its counterpart on the gray/white matter surface because of the one-to-one correspondence) as the mean of the two shortest distances (Fischl and Dale, 2000); one was from the point on the pial surface to the gray/white surface, and the other was from the point on the gray/white matter surface to the pial surface. Vertex-wise estimates of the surface area were calculated by assigning one-third of the area of each triangle to each of its vertices (Wang et al., 2014).

### lGI

After obtaining the pial surface, a cortical map of lGI can be obtained in three main steps (Schaer et al., 2008). First, an outer surface composed of vertices and triangles was generated by triangulating the outer hull that tightly wraps the pial surface. Second, the lGI is computed for each of the vertices of the outer surface, as a ratio of areas of circular region centered on this vertex and the area of the corresponding region on the pial surface. Third, a cortical map of lGI is obtained by propagating the lGI values from the outer surface mesh to the pial surface.

### Resampling and Smoothing

Before group comparison, all of the individual reconstructed cortical surfaces were aligned to an average template by using a surface-based registration algorithm (Fischl et al., 1999b). Then the cortical thickness, surface area and lGI maps were resampled. Finally, to increase the signal-to noise ratio and improve the ability to detect morphometric variations, we further smoothed the resampled cortical thickness, surface area maps and lGI map with a heat kernel of 20 mm (Wang et al., 2014) and 10 mm width (Zhang et al., 2014), respectively.

### Statistical Analyses

The Mann-Whitney U test was used to analyze the differences in PHES test performance between the two groups. Analyses were conducted using commercially available statistical software (SPSS, version 16.0; Chicago, IL, USA), and a *p* value of less than 0.05 (two-tailed) was deemed significant.

Vertex-by-vertex contrasts of cortical thickness, surface area and lGI were performed between normal controls and patients with HBV-RC-NOHE. Specifically, each contrast was entered into a vertex-by-vertex GLM including diagnosis, sex and age as covariates. Subsequently, a corrected cluster-wise *P* value was obtained using random field theory (RFT; Hayasaka et al., 2004). The level of significance for vertices was set at a surface-wide *P* < 0.05 after multiple-comparison correction. Within the patient group, a partial correlation analysis was performed between the average cortical thickness, surface area, and lGI of each cluster of differences (COD) and PHES controlling sex and age. The level of significance for the correlation analyses was set at *P* < 0.05.

## Results

**Figure 1:**
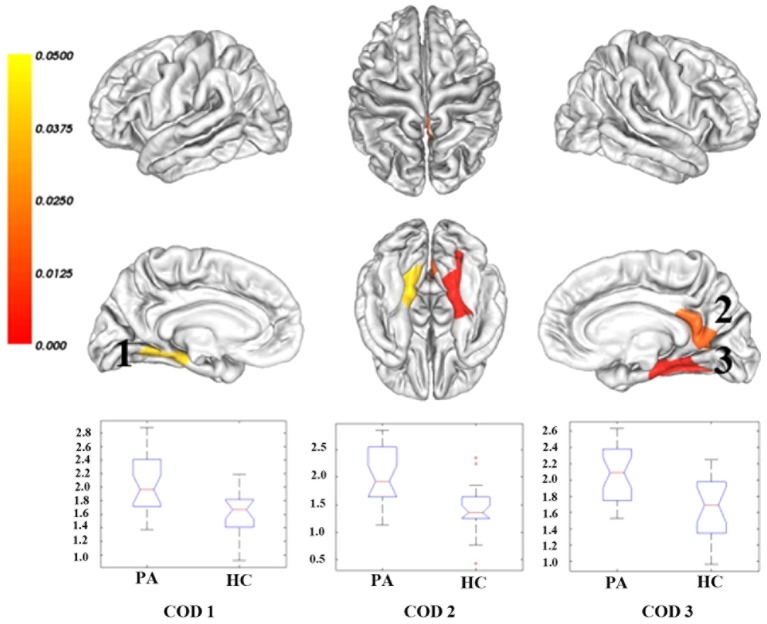
**Regions showing significantly increased cortical thickness in patients with HBV-RC-NOHE compared with healthy controls**. The results were corrected for multiple comparisons (*P* < 0.05, the cluster-based RFT correction). The color bar indicates the corrected *P* values. The integers are the COD IDs corresponding to those of Table 2. The distributions of the mean cortical thickness of each COD are displayed using notched plot boxes, and the outliers are marked using asterisks. Healthy controls, HC; Patients with HBV-RC-NOHE, PA.

**Figure 2:**
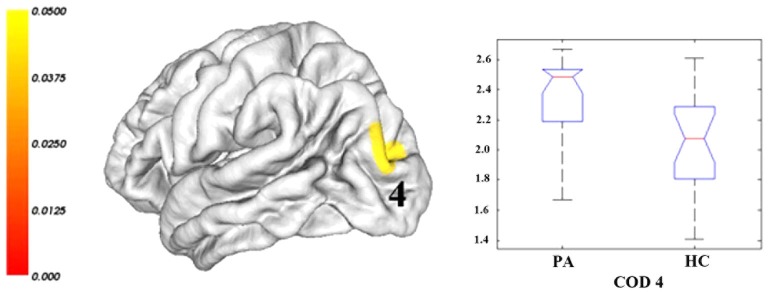
**Regions showing significantly increased lGI in patients with HBV-RC-NOHE compared with healthy controls**. The results were corrected for multiple comparisons (*P* < 0.05, the cluster-based RFT correction). The color bar indicates the corrected *P* values. The integer is the COD IDs corresponding to those of Table 2. The distribution of the mean lGI of COD 4 is displayed using notched plot boxes, and the outliers are marked using asterisks. Healthy controls, HC; Patients with HBV-RC-NOHE, PA.

Compared with healthy controls, cirrhotic patients performed worse on the PHES test (−3 [−9–1] for patients vs. 0 [−2–3] for controls; *P* < 0.001). Vertex-wise contrasts of the surface-based measurements revealed increased cortical thickness and gyrification in patients with HBV-RC-NOHE. Cortical thickness analysis found three CODs involving the bilateral lingual and parahippocampal gyrus, right posterior cingulate cortex, precuneus, pericalcarine sulcus and right fusiform gyrus (Figure 1; Table 2). Cortical gyrification analysis found only one COD involving the left superior and inferior parietal cortex as well as lateral occipital cortex (Figure 2; Table 2). No significant difference in surface area was observed between the two groups.

**Table 2:** **Regions showing significant cortical thickness/lGI increase in patients with HBV-RC-NOHE compared with healthy controls**.

COD IDs	Anatomic regions	Side	Cluster size (vertices)	Controls	Cirrhotic patients	Percentage increase	*P* value	Peak coordinates (*x, y, x*)
1	Lingual and parahippocampal gyrus	Left	1415	1.62 (0.31)	2.03 (0.41)	25.3%	0.0406	−20.9, −46.2, −11.2
2	Posterior cingulate cortex; precuneus; Pericalcarine sulcus	Right	3061	1.43 (0.42)	2.00 (0.57)	39.9%	0.0153	22.1, −63.1, 6.5
3	Lingual and fusiform gyrus; Parahippocampal gyrus	Right	3723	1.66 (0.36)	2.07 (0.34)	24.7%	0.0028	34.5, −42.6, −12.9
4	Inferior and superior parietal cortex; Lateral occipital cortex	Left	1497	2.05 (0.34)	2.37 (0.23)	15.6%	0.0443	−30.4, −82.4, 14.7

**Figure 3:**
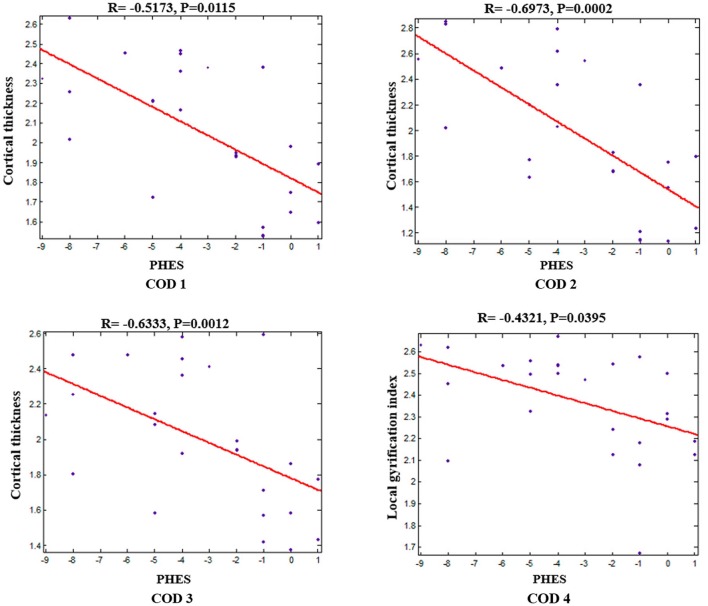
**Negative correlations between mean cortical thickness/lGI of each COD and PHES in patients with HBV-RC-NOHE while adjusting for sex and age**.

Within the patients group, correlation analyses revealed significant negative correlations between the average cortical thickness/lGI of the corresponding COD and PHES (correlations between cortical thickness and PHES: *r* = −0.5173, *p* = 0.0115 for COD 1; *r* = −0.6973, *p* = 0.0002 for COD 2; *r* = −0.6333, *p* = 0.0012 for COD 3; correlation between lGI and PHES: *r* = −0.432, *p* = 0.0395 for COD 4; Figure 3).

## Discussion

In the current study, surface-based morphometry was applied to quantify the cortical thickness, area and gyrification in well-matched samples of patients with HBV-RC-NOHE vs. healthy controls. We observed increased cortical thickness and lGI in patients with HBV-RC-NOHE. In addition, we also revealed negative correlations between the mean cortical thickness/lGI of each COD and PHES.

### Cortical Thickness

In the present study, we observed significantly increased cortical thickness in patients with HBV-RC-NOHE. The regions showing significantly increased cortical thickness are functionally relevant to the neurocognitive deficits in patients with HBV-RC-NOHE. Specifically, the lingual gyrus, calcarine sulcus, fusiform gyrus and parahippocampal gyrus are key nodes of ventral visual pathway, abnormalities in which may relate to impaired visual information processing in cirrhotic patients. The posterior cingulate cortex and precuneus have reciprocal connections with other parietal areas, such as the operculum, and inferior and superior regions of the lateral posterior parietal lobe (Selemon and Goldman-Rakic, 1988; Cavada and Goldman-Rakic, 1989), which were involved in visuospatial information processing (Ungerleider and Haxby, 1994; Corbetta et al., 1995). Hence, the abnormalities in the posterior cingulate cortex and precuneus might be related to the visuospatial deficits in cirrhotic patients. These suggestions are supported by the observed negative correlations between the mean cortical thickness of three CODs and the PHES.

The observed cortical thickness increase in cirrhotic patients seems to contradict a previous report of cortical thickness reduction in patients with MHE (Montoliu et al., 2012). The exact cause of such inconsistency remains unclear. It possibly relates to differences in the demographic characteristics of patients groups. In their study, the sample of the patient group included cirrhotic patients with various causes, most of which were alcohol-related cirrhosis. Given earlier studies have confirmed that alcohol can cause brain atrophy (Sutherland et al., 2014), the cortical thickness reduction in their study may be due to the effect of alcoholism. On the other hand, such inconsistency may also arise from differences in the severity or the pathological stage of the patient group, since cirrhotic patients of different disease stages may exhibit different patterns of structural abnormalities (Guevara et al., 2011; Zhang et al., 2012).

Given that cortical thickness measures the size, density and arrangement of cells, cortical thickness alterations may correspond to substantial pathological changes in the underlying cell counts and organization (Narr et al., 2005; Zhang et al., 2011). The exact mechanism underlying the cortical thickness increase in patients with HBV-RC-NOHE remains unclear. However, given previous studies have documented thicker cortical thickness in the central sulcus in the presence of vasogenic edema (Biega et al., 2006; Togao et al., 2008), it is tempting to speculate that the cortical thickness increase relates to low-grade brain edema in patients with chronic cirrhosis (Häussinger et al., 2000; Bosoi and Rose, 2013). This speculation is further supported by a recent study which indicated that a decline in water content of the brain could produce a thinner cortex (Bansal et al., 2013). In patients with cirrhosis, the neurotoxic effects of ammonia have long been considered the main pathogenic factor via astrocyte swelling and cerebral edema (Atluri et al., 2011). Indeed, astrocytes are the only cell compartment in the brain containing glutamine synthetase and are accordingly the major site of cerebral ammonia detoxification (Häussinger et al., 2000). In states of hyperammonemia, ammonia detoxification within astrocytes leads to an intracellular accumulation of glutamine (Aldridge et al., 2015), which could result in a re-establishment of the osmotic equilibrium and, consequently, an increase in brain water uptake and vasogenic brain edema in patients with chronic cirrhosis (Bosoi and Rose, 2013). Therefore, the observed increase in cortical thickness might be closely related to altered intracortical organization due to the low-grade brain edema in patients with cirrhosis.

### lGI

Compared with controls, patients with HBV-RC-NOHE showed greater gyrification in the left inferior and superior parietal cortex as well as lateral occipital cortex. The inferior and superior parietal cortex as well as the lateral occipital cortex are key nodes of the dorsal visual pathway, which is generally accepted to be responsible for motion perception, spatial awareness, and vision for action (Laycock et al., 2011). Moreover, the lateral posterior parietal cortex including the inferior and superior parietal cortex was considered as an important hub where different types of information, such as sensory, motor, goal-related and reward, are integrated (Singh-Curry and Husain, 2009). Hence, the cortical gyrification changes in this region may relate to dysfunction in visuospatial and visuomotor integration in patients with HBV-RC-NOHE. This speculation is also supported by the observed negative correlation between the PHES and the mean lGI of this region.

The exact mechanism underlying the cortical gyrification changes is not clear, although a number of theories about the ontogenesis of cortical gyrification have surfaced (White et al., 2010; Zilles et al., 2013). Among these theories, the most widely accepted explanation for the gyrification changes is a neuromechanical hypothesis that cortical gyrification is mainly due to the tension of the underlying white matter connectivity (Van Essen, 1997; Herculano-Houzel et al., 2010). In fact, both structural and functional connectivities disturbances have been observed in cirrhotic patients (Qi et al., 2012; Chen et al., 2014). Moreover, widespread alterations in mean diffusivity and fractional anisotropy as well as focal changes in white matter volume have been reported in cirrhotic patients (Kale et al., 2006; Kumar et al., 2008; Chen et al., 2012; Qi et al., 2013). Hence, the cortical gyrification increase may be closely related to the disturbed white matter pathways. Besides, another mechanical model of brain convolutional development has also been used to explain abnormalities in cortical gyrification. This model proposes that differential growth rates of cortical layers directly affect the degree of cortical convolutions (Caviness, 1975). Although no direct evidence of abnormal cortical layer growth rates in HBV-RC-NOHE were provided in the present study, the aforementioned swelling of the astrocytes secondary to hyperammonemia is indicative of suboptimal intracortical organization (Häussinger et al., 2000), which may account for the cortical gyrification abnormalities observed in patients with HBV-RC-NOHE.

### Correlations with PHES

A novel finding in this study was the identification of negative correlations between performance on PHES and the mean cortical thickness/lGI of the CODs, suggesting that the more serious the cognitive impairments, the more obvious is the dysfunction detected in those regions. Our findings further suggest that the visual-related cortical dysfunction might be involved in the pathophysiology of deficits in visual processing in cirrhotic patients. In addition, the correlations between the cortical thickness/lGI and the PHES indicate that such indices in these brain regions may serve as potential biomarkers to reflect the severity of cognitive changes in patients with HBV-RC-NOHE.

### Limitations

There are several limitations that should be addressed. First, as a preliminary study, our results are limited to a small patient cohort, which may have an effect on the power of the statistical analysis of this study. Further studies with more patients are needed to replicate the current findings. Second, given the nature of cross-sectional study, the present study does not allow for the investigation of the dynamic development of the structural abnormalities in these patients from HBV-RC-NOHE to OHE. Further studies adopting a longitudinal design is needed to unveil the dynamic pattern of the structural abnormalities as the disease progresses.

In conclusion, the present study performed a comprehensive analysis on the cortical morphometry of patient with HBV-RC-NOHE. Compared with healthy controls, we found increased cortical thickness and gyrification in patients with HBV-RC-NOHE. In addition, we revealed significant negative correlations between the mean cortical thickness/lGI of each COD and PHES in patients with HBV-RC-NOHE. These morphological changes may serve as potential markers for the preclinical diagnosis and progression of HBV-RC-NOHE.

## Conflict of Interest Statement

The authors declare that the research was conducted in the absence of any commercial or financial relationships that could be construed as a potential conflict of interest.

## Footnotes

^1^http://surfer.nmr.mgh.harvard.edu/
